# Acupuncture for rehabilitation after total knee arthroplasty: a systematic review and network meta-analysis

**DOI:** 10.1097/JS9.0000000000002006

**Published:** 2024-08-05

**Authors:** Wen-Xuan Li, Cai-Qin Wu, Wei Feng, Yi-Jun Zhan, Lei Yang, Heng-Jie Jia, Jian Pei, Kun-Peng Li

**Affiliations:** a Longhua Hospital Affiliated to Shanghai University of Traditional Chinese Medicine, Shanghai, China; b The Second Rehabilitation Hospital of Shanghai, Shanghai, China; c School of Acupuncture-Moxibustion and Tuina, Shanghai University of Traditional Chinese Medicine, Shanghai, China; d School of Nursing, Shanghai University of Traditional Chinese Medicine, Shanghai, China; e School of Electronic Information and Electrical Engineering, Shanghai Jiao Tong University, Shanghai, China; f Shanghai Jiao Tong University School of Medicine, Shanghai, China; g School of Exercise and Health, Shanghai University of Sport, Shanghai, China

**Keywords:** acupuncture, network meta-analysis, total knee arthroplasty

## Abstract

**Background:**

The increasing interest in acupuncture for promoting postoperative rehabilitation has encouraged its use in rehabilitation after total knee arthroplasty (TKA), but its effectiveness remains controversial.

**Objective:**

This study aims to assess the efficacy of different models of acupuncture-related therapies on pain relief, motor function, inflammation, and overall rehabilitation of the knee after TKA.

**Methods:**

Databases PubMed, Embase, Cochrane, Web of Science, Scopus, and Clinicaltrials.gov were searched to screen eligible randomized controlled trials (RCTs). All RCTs that used acupuncture/moxibustion on TKA patients were included by two researchers independently after rigorous quality evaluation, with data extracted. The statistics were performed by using R 4.2.3 and Stata 17.

**Results:**

The network meta-analysis incorporated 28 RCTs, 21 of which were conducted in mainland China and Taiwan. Evidence of the relation of several common acupuncture/moxibustion treatments was shown by the network meta-analysis (NMA). The results of NMA showed that electroacupuncture was the preferred therapy for soothing pain symptoms (standardized mean difference=0.58, 95% CI=0.36–0.81). Auricular acupressure was the best treatment to improve the knee motor function. Electroacupuncture was superior to special acupuncture in relieving knee joint stiffness and inflammation.

**Conclusion:**

Acupuncture intervention, especially electroacupuncture, can effectively alleviate pain, improve motion, reduce stiffness, and decrease inflammation in post-TKA patients with no serious adverse events.

## Introduction

Highlights

- A network meta-analysis was innovatively introduced to evaluate the effectiveness of different forms of acupuncture-related therapies on pain relief, motor function, inflammation, and overall post-TKA rehabilitation.
- The effectiveness of acupuncture in both knee osteoarthritis and rehabilitation after total knee arthroplasty was comprehensively considered.
- All acupuncture-related treatments were innovatively integrated on the basis of previous studies to provide more clinically instructive conclusions.

The global prevalence of knee osteoarthritis (KOA) stands at ~16.0%, with an incidence rate of 203 per 10 000 individuals^1^. Total knee arthroplasty (TKA) represents the established standard procedure for alleviating the challenges posed by advanced KOA^2,3^. In the United Kingdom alone, the annual count of knee replacements has exceeded 100 000^4^. Projections indicated an 85% surge in first-time TKAs in the United States by 2030, reaching 1.26 million procedures, compared to 2014 figures^5^. However, the presence of postoperative pain, hematoma, swelling, and compromised quadriceps muscle strength^6,7^ presents significant barriers to effective knee rehabilitation. Notably, a study revealed that patients experienced a 20–25% decline in muscle function one month post-TKA^8^. Even after one year, patients exhibited lower muscle function compared to healthy adults, with a slowdown of 18% in walking speed and 51% in climbing speed^9^. Consequently, dissatisfaction with rehabilitation outcomes following TKA was reported by 15–20% of patients^10^. Thus, comprehensive strategies addressing the postoperative complications associated with TKA are imperative.

Several guidelines^2,11–14^ have recommended treatment approaches for KOA based on pain, function, and disability assessment, advocating for aerobic exercise, education, and the use of topical non-steroidal anti-inflammatory drugs. In the context of post-TKA rehabilitation, non-pharmacological interventions, such as preoperative exercise^15^ and cryotherapy^16^, have gained considerable attention. These approaches aim to enhance rehabilitation outcomes and minimize the risk of adverse drug reactions in certain patients. Acupuncture has been frequently employed as a standalone or adjunctive therapy when the aforementioned measures yield limited effects^17,18^. It has shown efficacy as a treatment modality for KOA^19^, particularly in patients experiencing moderate to severe pain who are either unwilling or ineligible for TKA surgery^14^. Some studies have also reported positive effects of acupuncture in reducing knee swelling^20^ and pain^21^ and facilitating early range of motion recovery^22^. However, AAOS^2^ has deemed evidence supporting acupuncture inconclusive, while EULAR^12^ and NICE^13^ do not recommend its use. Studies have even indicated that acupuncture has a significant placebo effect^23^ and does not positively impact recovery in osteoarthritis^24^, leading to discrepancies in the available clinical data and conclusions that necessitate further confirmation and consolidation. It is important to note that different acupuncture techniques carry distinct advantages, disadvantages, and potential adverse effects, and determining the optimal treatment approach remains elusive. Currently, comprehensive meta-analyses comparing the efficacy of various acupuncture methods in postoperative TKA rehabilitation are lacking, as is high-quality evidence from evidence-based medicine regarding the optimal acupuncture treatment regimen.

Hence, this study employed a network meta-analysis (NMA) to appraise the efficacy of diverse acupuncture-related therapies in facilitating the recovery of various physiological functions among individuals undergoing TKA. Specifically, this study focused on assessing the effectiveness in alleviating pain, enhancing motor function, reducing inflammation, and promoting overall knee rehabilitation subsequent to TKA. By doing so, this research aimed to furnish clinicians and evidence-based medicine researchers with robust evidence to facilitate informed treatment decisions in the postoperative rehabilitation of TKA patients.

## Methods

The study protocol was pre-registered in the PROSPERO database and implemented in strict adherence to PRISMA (Supplemental Digital Content 1, http://links.lww.com/JS9/D249) (extension statement for network meta-analysis)^25^ and AMSTAR (Supplemental Digital Content 2, http://links.lww.com/JS9/D250) (Assessing the methodological quality of systematic reviews)^26^ Guidelines.

### Search strategy

An information specialist developed the search strategy and searched six electronic databases: PubMed, Embase, Cochrane, Web of Science, and Scopus with Full Text, from the inception date to 10 June 2023. In addition, one trial registry, ClinicalTrials.Gov, was searched from its inception date to June 2023 to identify registered trials and relevant reports. No restrictions were applied on the date, country of origin, or publication status.

The combination of the following keywords was employed for the PubMed search: (“acup*” OR “needl*” OR “pharmacopunctur*” OR “electroacupuncture*” OR “moxibustion”) AND (“arthroplasty, replacement, knee” OR “total knee arthroplasty” OR “total knee replacement” OR “TKA”) AND (“randomized controlled trial” OR “controlled clinical trial” OR “randomized” OR “placebo” OR “randomly” OR “trial”). Queries were revised to perform the best searches in the other databases. The search strings for various databases were presented in Supplementary Material (Supplemental Digital Content 3, http://links.lww.com/JS9/D251 and Supplemental Digital Content 4, http://links.lww.com/JS9/D252).

### Study selection

After exporting the references and removing duplicates, titles, and abstracts of records, they were screened independently by two reviewers according to the following eligibility criteria. Full texts of all potentially relevant trials were subsequently retrieved and reviewed to confirm the final eligible trials. Any disagreements were resolved via consensus, and when any disagreement was elusive, a third reviewer acted as an arbiter.

### Eligibility criteria

(1) Only randomized controlled trials (RCTs) were considered. (2) Only studies which investigated patients undergoing TKA were accessed, irrespective of gender, age, and disease duration. (3) In addition to routine treatment after TKA, the experimental group received acupuncture treatment alone or in combination with other therapies, while the control group did not receive acupuncture or received sham acupuncture. (4) Only studies reporting the pain and/or functional outcomes of patients were considered for inclusion. Missing data under the outcomes of interest warranted exclusion from this study. (5) Articles in both English and Chinese were eligible.

### Data extraction

A total of 28 studies meeting the eligibility criteria underwent rigorous review and coding by one primary reviewer, with confirmation provided by a second reviewer. In cases where discrepancies arose, a third reviewer was consulted to reach a resolution. The data extraction process encompassed various aspects, including authors’ names, publication year, country of origin, patient demographics such as age and gender, sample size, intervention type, acupoints utilized, needle retaining time, intervention dosage, and primary outcome measures.

For the analysis, the means (M) and standard deviations (SD) of all relevant baseline and post-intervention reactive balance measures were extracted. In instances where data were missing, specifically pertaining to eligibility criteria or study outcomes, that were not reported either in the text or on publicly accessible data repositories, corresponding authors were contacted via e-mail to provide the necessary information. A follow-up request was issued if no response was received within one month, and if no reply was received within an additional month, the data were deemed irretrievable.

### Risk of bias

To evaluate the risk of bias at both the overall and study level, a team of two reviewers independently assessed potential biases in the trials. The assessment was conducted using the Cochrane risk of bias tool (RoB 2): (1) randomization process; (2) deviations from the intended interventions; (3) missing outcome data; (4) measurement of the outcome; and (5) selection of the reported result^27^. Each domain was assigned a judgment of “low risk”, “some concerns”, or “high risk”. Disagreements were resolved through discussion or referral to a third reviewer.

### Statistical analysis

Given the uncertainty regarding the baseline similarity of outcome measurements in several studies, the change values from baseline to post-intervention were calculated or directly extracted from published data. If there were multiple post-intervention measures (e.g. post-intervention and follow-up) or multiple time points for outcome indicators, only the data from the end of the intervention phase were used. The missing SDs are derived from standard error (SE) and 95% confidence intervals (CI). The network package of Stata 17 software is used to calculate the standardized mean difference (SMD) and standard error (SE) and analyze the publication bias.

To assess the relative effectiveness of acupuncture-based interventions in post-TKA rehabilitation, an NMA was conducted that incorporated both direct (i.e. positive comparisons derived from pairwise meta-analyses) and indirect comparisons (i.e. derived from NMA) within a statistical model. The NMA was performed within a Bayesian framework using Markov Chain Monte Carlo simulations, with non-informative prior distributions employed to handle the treatment effects^28,29^. Given the clinical and methodological heterogeneity across studies, a random effects model was first employed^30,31^. The NMA included all available acupuncture interventions from at least two trials.

The overall geometric shape of a network was represented in the form of a network graph. Based on the Bayesian posterior rank probabilities, the ranking of acupuncture interventions was measured using the rank probability graph. The standardized mean difference (SMD) posterior distribution was reported using the average difference of the reference intervention with a 95% credible interval (CrI), which indicated that there was a 95% probability that the unobserved (unknown) effect estimate falls within the interval^32^. If the 95% CrI includes zero (i.e. the null effect representing the null hypothesis), the effect can be considered statistically non-significant^32^. The relative effects of all acupuncture interventions were reported in the matrix with their 95% CrI. The analysis involved a burn-in period of 50 000 iterations and a follow-up period of 100 000 iterations to minimize the bias of initial values when the chains reached their target distributions^33^. The convergence of the model was assessed using trace plots, density plots, and the Brooks–Gelman–Rubin diagnostic statistic^33^. Consistency is a crucial assumption in network meta-analysis (NMA), indicating the agreement between direct and indirect estimates within the network^34^. This study used node-splitting analysis to determine whether to apply a consistency or inconsistency model. In situations without indirect comparisons, the consistency model could be directly applied for analysis. All data synthesis and statistical analyses were conducted using the “Gemtc” package (version 1.0-2), “rjags” package (version 4-14), and “netmeta” package (version 2.8-2) in R software (version 4.2.3, R Foundation for Statistical Computing, Vienna, Austria).

## Results

### Study selection

**Figure 1:**
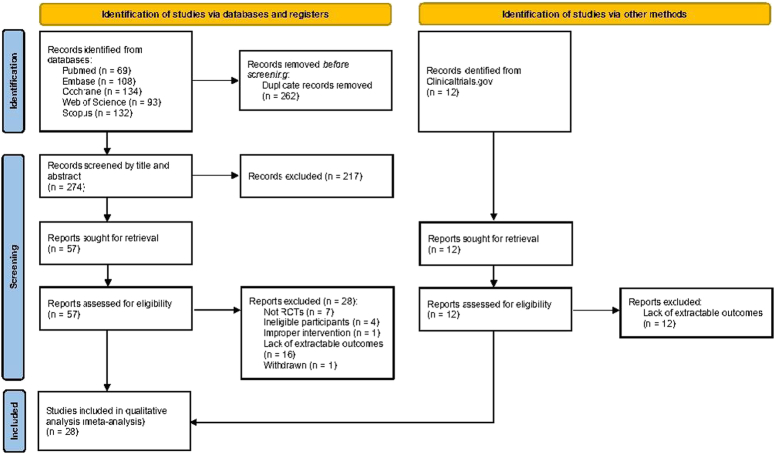
Study selection flow diagram.

A total of 536 records were retrieved from electronic databases, and an additional 12 records were retrieved from other sources. After removing duplicates and screening titles and abstracts, 57 research reports remained. Based on full-text screening, 28 records met the inclusion criteria and were included in the qualitative analysis (i.e. systematic review). The schematic flowchart for the selection process is presented in Figure 1.

### Characteristics of included studies

Of the 28 RCTs included in the network meta-analysis, 21 were conducted in the Chinese mainland and Taiwan region. The eligible studies represented a total of 2128 participants, included in both pre-intervention and post-intervention analyses, with the ages ranging from 55.8 to 73.2 years. In all included studies, the mean age of the acupuncture group ranged from 55.8 to 73.1 years. The age of the control group ranged from 60.1 to 73.2 years, and the difference between the two groups was small. Participants were evenly proportioned in terms of gender (407 men in the acupuncture group versus 402 in the control group). Table 1 presents the characteristics of all the participants of the included studies.

**Table 1:** Characteristics of all the participants of the included studies

References	Country	BMI		Age		Sample size		Gender (male)		Dropout	
		A	C	A	C	A	C	A	C	A	C
Cao Hai-tao^35^	China	23.6±3.6	22.8±4.8	66.3±7.3	66.4±8.5	44	45	11	13	3	2
Chang^36^	Taiwan, China	27.19±3.08	28.73±4.14	71.23±7.09	70.74±8.09	31	31	3	6	0	0
Chen^37^	China	—	—	67.4±5.1	65.6±5.1	35	35	13	12	0	0
Chen^38^	Taiwan, China	—	—	68.9±9.0	69.0±8.6	31	31	27	22	1	1
Chen^39^	China	—	—	72±6	70±5	60	60	18	22	0	0
Chen^40^	China	—	—	55.80±15.75	56.93±14.61	46	46	32	36	0	0
Durmus^41^	Turkey	30.92±5.13	32.6±5.03	65.37±5.9	66.84±8.89	30	31	2	2	0	0
He^42^	China	—	—	62.56±6.10	61.58±6.66	45	45	18	16	0	0
Huang^43^	China Taiwan	—	—	73.10±7.37	73.20±8.28	39	40	13	15	2	1
Huang^44^	Taiwan, China	26.79±3.91	28.52±5.21	73.10±7.37	73.20±8.28	39	40	13	15	0	0
Ju^45^	China	26.34±2.14	26.34±2.14	65±6	65±6	87	0	33	35	0	0
Kang^46^	China	25.74±2.83	25.94±3.04	70.19±5.42	70.46±4.9	63	63	9	7	3	3
Kang^47^	China	25±1.9	25.7±1.9	71.4±6.1	69.4±5	16	15	2	2	0	0
Kim^48^	South Korea	24.52±3.45	25.60±3.45	63.53±4.29	62.07±3.88	15	15	0	0	0	0
Ma^49^	China	22.99±3.82	22.01±3.50	69.63±7.60	70.92±6.55	41	41	—	—	3	2
Mavrommatis^50^	Greece	30.5±4.6	31.8±4.7	62.3±9.9	60.1±11.1	39	39	27	31	1	1
Mayoral^51^	Spain	73.57±11.53	75.51±9.33	71.65±6.06	72.9±7.85	20	20	11	0	20	20
Mikashima^52^	Japan	—	—	72±7	73±5	40	40	10	12	0	0
Petersen^53^	Denmark	—	—	56±8	56±6.8	87	85	38	33	0	0
Ren^54^	China	—	—	65.61±7.42	64.06±8.65	69	67	20	23	0	0
Shi^55^	China	—	—	66.7±4.6	67.0±4.9	40	40	18	16	0	0
Si^56^	China	—	—	65.5±6.1	64.7±6.7	20	20	11	14	0	0
Tao^57^	China	—	—	71±11	68±10	40	40	23	21	0	0
Tong^58^	China	—	—	70±4.79	71.37±3.1	30	30	11	12	0	0
Tsang^59^	Singapore	29.7±3.9	27.6±5	70.6±5.8	66.1±7.5	18	18	6	0	6	
Tzeng^60^	Taiwan, China	27.41±0.23	28.48±0.2	69.6±5.6	70.1±6.9	16	17	4	3	0	0
Zhang^61^	China	24.81±3.34	23.51±4.78	66±7	63±7	37	36	22	20	2	1
Zhao^62^	China	—	—	65.23±4.03	66.70±3.84	30	30	12	14	0	0

A, acupuncture-related therapies group; C, conventional rehabilitation group.

The main features of the 28 included studies are summarized in Table 2. The intervention measures involved essentially encompassed all clinically common acupuncture therapies currently employed^63^. Besides conventional acupuncture, wrist–ankle acupuncture and auricular acupressure are acupuncture focusing on special parts of the human body. Electroacupuncture, laser acupuncture, and transcutaneous electrical nerve stimulation (TENS) are the application of electric current or laser to conventional acupuncture to enhance the stimulation intensity. Dry needling usually acts on trigger points, which is different from the acupoints of conventional acupuncture. Warm acupuncture involves wrapping moxa wool around the tail of the needle and then burning it, which makes it different from moxibustion^64^.

**Table 2:** Main features of all the included studies

References	Intervention							Outcome		Adverse Events		
	Type		Start time		Acupoints	Retaining time	Dosage	Indicator	Time points for evaluation (after operation)	Type	A	C
	A	C	A	C								
Cao Hai-tao^35^	Wrist–ankle acupuncture	Sham	First day after surgery		Ashi points	30 min	1/day, 1 week	VAS, ROM, active straight-leg raising time	1, 2, 3 days	Minor pain and bleeding	—	—
Chang^36^	Auricular acupressure	Sham	First day after surgery		Shenmen and subcortex	3 min	3/day, 3 days	VAS, SF-MPQ, Pain PPI	1, 2, 3 days	None		
Chen^37^	Electroacupuncture	Routine rehabilitation therapy	5 days to 2 weeks after surgery	1st day after surgery	Xuehai (SP10), Liangqiu (ST34), Dubi (ST35), Neixiyan (EX‐LE 4) and Yanglingquan (GB34), etc.	30 min	First 2 weeks: 1/day; 3–12 weeks: 2/week	HSS, ROM, MMT, VAS	2, 6, 12 weeks	—		
Chen^38^	Auricular acupuncture and electroacupuncture	Sham auricular acupuncture	Anesthetized and right before surgery started		Xuehai (SP10), Liangqiu (ST34), Weizhong (BL40), Xiguan (LR7), Zusanli (ST36); Sensory area of the scalp acupuncture, Baihui (GV20), and Shenting (GV24); Chinese auricular acupoints: ear Shenmen (shemen, TF4), knee point (xi, AH4), sympathesis point (jiaogan, AH6a), and subcortex point (pizhixia, AT4)	20 min	1 session	VAS	2, 4, 8, 12, 24, 36, and 48 h	Nausea/vomiting	3	15
										Respiratory depression	0	1
										Hypotension	1	7
										Urine retention	2	7
										Pruritus	1	3
										Sedation	1	1
Chen^39^	Acupoint massage and mild moxibustion	Routine detumescence	Acupuncture: 1 h after surgery; mild moxibustion: 48 h after surgery		Zusanli (ST36) and Sanyinjiao (SP 6)	till soreness and distension presented	Acupoint massage: 2/day, 2 days; moxibustion: 1/day, 5 days	VAS, HSS	1, 3, 5, 7, and 14 days	None		
Chen^40^	Electroacupuncture	Acupuncture	1 h after surgery		Taichong (LR 3), Yang Lingquan (GB 34), Waiguan (TE 5), and Chize (LU 5)	30 min	Every 12 h for 3 days after surgery	VAS	2 h, 1, 2, 3 days	—	A lower than C	
Durmus^41^	Acupressure	Control	5 days after surgery		Tianshu (ST25), Zhongwan (CV12), and *TB6	3 min	Once a day – five séances in total	VAS, DCFF	First and second defecation	—		
He^42^	Auricular acupressure	Four nonacupuncture points of the helix ipsilateral	—		Auricular acupoints: knee joint, shenmen, subcortex, sympathesis	3 min	4/day, 1 week	VAS, dosage of the used analgesic via PCA, HSS, ROM	12 h, and 1, 2, 3, 4, 5, 7 days, and 2 weeks, and 3 months	Nausea and vomiting	8	32
										Dizziness and drowsiness	2	11
										Uroschesis	3	12
Huang^43^	Laser acupuncture	Sham ALLLT at the same acupuncture points without using the laser beam	2 h after surgery		Neiguan (PC6), Sanyinjiao(SP6), Taixi (KI3), Kunlun (BL60), Fengshi (GB31), and Futu (LI18)	10 s	Second hour, sixth hour, 10th hour, day 1, day 2, and day 3 postoperatively	Knee joint flexion, WOMAC stiffness	1, 2, 3 days	None		
Huang^44^	Low-level laser acupuncture	Same treatment procedure without laser energy output	2 h after surgery		Sanyinjiao (SP6), Taixi (KI3), Kunlun (BL60), Fengshi (GB31), Futu (ST32), and Neiguan (PC6)	2, 6, 10, 24, 48, and 72 h after surgery		BPI, morphine consumption	2, 6, 10 h, and 1, 2, 3 days	Nausea/vomiting	0	4
										Sedation	0	1
										Dizziness	0	3
Ju^45^	Warm acupuncture	Exercise	1st day after surgery	1–14 days after surgery	Liangqiu (ST34) and Zusanli (ST36)	15 min	2/day, 2 weeks	VAS, average first straight-leg raising time, average time of the first time for standing up	1, 2, 3, 4 days	None		
Kang^46^	Electroacupuncture	Sham acupuncture	3rd day after surgery		Futu (ST32), Zusanli (ST36), Yinlingquan (SP9), Yanglingquan (GB34)	20 min	1/day, 5 days	—	3 days	None		
Kang^47^	Electroacupuncture	Noninserted sham needles	3rd day after the surgery		Futu (ST32), Zusanli (ST36), Yinlingquan (SP9), and Yanglingquan (GB34)	20 min	1/day, 5 days	NRS, SDS	—	None		
Kim^48^	Acupuncture-like transcutaneous electrical nerve stimulation (TENS)	Conventional TENS	—		Yinmen (BL37), Futu (ST32), Liangqiu (ST34), Xuehai (SP10)	30 min	5/week, 2 wk	VAS, TUG, WOMAC stiffness, WOMAC pain, WOMAC function, WOMAC total, CRP level	At rest, after TUG	—		
Ma^49^	Electroacupuncture	Routine postoperative analgesia program	1st day after surgery		Shousanli (LI10), Quchi (LI11), Zhouliao (LI12), Binao (LI14), Chize (LU5), Sidu (SJ9)	20 min	1/day, 5 days	VAS, tenderness threshold, number of pressing times of patient‐controlled analgesia pump, dose of extra analgesics, HSS, HADS	2, 3, 5 days	None		
Mavrommatis^50^	Acupuncture and electroacupuncture starting from the third session	Sham acupuncture	More than 3 months		Zusanli (ST36), Yinlingquan (SP9), Xuehai (SP10), Yanglingquan (GB34), Heding (Ex-LE2), and Xiyan (Ex-LE5); Hegu (LI4), Taixi (KI3), Fenglong (ST40), and Sanyinjiao (SP6)	20 min	Biweekly for 8 weeks	WOMAC, VAS, algometer, creatinine, AST, ALT	4, 8, 12 weeks	—		
Mayoral^51^	Dry needling	Sham dry needling	Immediately after each subject was anesthetized and right before surgery started		Myofascial trigger points	—		VAS, WOMAC, ROM, Strength FLEX, Strength EXT	1, 3, 6 months	—		
Mikashima^52^	Acupuncture	Sham	1 week after surgery		Biguan (ST31), Futu (ST32), Tiaokou (ST38); Sanyinjiao (SP6); Shenshu (BL23), Dachangshu (BL25), Yinmen (BL37), Chengshan (BL57), Kunlun (BL60); Taixi (KI3); Fengshi (GB31), Xuanzhong (GB39), Qiuxu (GB40), Zulinqi (GB41), Diwuhui (GB42); Taichong (LR3)	20–30 min	3/week, 2 weeks	VAS, ROM, swelling around the knee	—	Fewer		
Petersen^53^	Acupuncture and exercise	Exercise	3 weeks after surgery		Futu (ST32) and Fengshi (GB31); Xuanzhong (GB39), Jiexi (ST41), and Taichong (LR3); Xuehai (SP10), Liangqiu (ST34), Ququan (LR8), Yinlingquan (SP9), and Zusanli (ST36)	15–20 min	2/week, 6 weeks	KOOS, AROM-Ext, walking distance	Post-intervention, 3 months	Did not appear to have a higher risk of adverse effects		
Ren^54^	Moxibustion	Sham moxibustion	More than 3 months		Dubi (ST35), Neixiyan (EX-LE4), and an Ashi point	20 min	3/week, 6 weeks	SF-36	3, 6, 12 weeks	None		
Shi^55^	Electroacupuncture and celecoxib	Celecoxib	On the day of the operation		Waiqiu (GB36), Jinmen (BL63), *EX-LE29, Zusanli (ST36), Liangqiu (ST34), Xuehai (SP10), Diji (SP8), Yanglingquan (GB34)	30 min	1/day, 3 days	VAS, SRSS, IL-6, IL-10, TNF-α	1, 2, 3 days	Few		
Si^56^	Transcutaneous electrical acupoint stimulation	Without electrical stimulation	Before anesthesia, 8, 16, 36, and 56 h postoperatively		Shenmen (HT7)	30 min	8, 16, 36, and 56 h postoperatively	VAS, the quadriceps maximum voluntary isometric contraction	12 h and 1, 2, 3 days	—		
Tao^57^	Electroacupuncture	Combined spinal and epidural anesthesia and postoperative epidural analgesia	During the surgery and 1 month after surgery	—	Hegu (LI4), Qihai (CV6), Zhongwan (CV12), Pishu (BL20), Shenshu (BL23)	15 min	Every other day, 1 month	Insulin resistance	—	—		
Tong^58^	Auricular acupressure	General anesthesia	30 min after surgery		Chinese auricular acupoints: ear Shenmen (shemen, TF4), sympathesis point (jiaogan, AH6a), and subcortex point (pizhixia, AT4)	30 min	1/30 min	VAS, ROM	6 h, and 2, 3 days, and 2 weeks	—		
Tsang^59^	Acupuncture	Sham acupuncture	4 days after surgery		ST32 (Futu), ST33 (Yinshi), GB31 (Fengshi), GB35 (Yangjiao), GB34 (Yanglingquan), and ST36 (Zusanli)	20 min	—	NRS, TUG	4, 5, 6, 7, 8, 11, 12, 13, 14, 15 days	—		
Tzeng^60^	Electroacupuncture	PCA (1.5 μg/ml fentanyl and 0.1% bupivacaine)	1 day after surgery		Zusanli (ST36) and Yanglingquan (GB34)	30 min	1/day, 2 days	Time to patients’ initial demand for PCA, dosage of PCA and VAS	2 days	Vomiting	8	10
Zhang^61^	Aconite-isolated moxibustion combined with rivaroxaban	Orally rivaroxaban tablets, 10 mg a time, once a day	—		Yongquan (KI1)	30 min	1/day, 2 weeks	Blood flow velocity of the deep femoral vein	—	—		
Zhao^62^	Electroacupuncture	Placebo acupuncture approach (Streitberger placebo needle)	5 days prior to the surgery		Sishencong (EX-HN1), Shenting (GV24), Baihui (GV20), Benshen (GB13), Hegu (LI4), Taichong (LR3)	30 min	1/day, 5 days	MMSE, IL-1β, TNF-α, S-100β	1 and 3 days	None		

A, acupuncture-related therapies group; ALLLT, acupuncture with low-level laser therapy; AROM-Ext, active range of movement in knee extension (extension deficit); BL, the Foot Taiyang Bladder Channel; BPI, Brief Pain Inventory; C, conventional rehabilitation group; CRP, C-reactive protein; CV, the Ren Channel; EX, extra points; GB, the Foot Shaoyang gallbladder Channel; GV, the Du Channel; HADS, hospital anxiety and depression scale score; HSS, Hospital for Special Surgery; HT, the Hand Shaoyin Heart Channel; KI, the Foot Shaoyin Kidney Channel; KOOS, Knee injury and Osteoarthritis and Outcome Score; LI, the Hand Yangming Large Intestine Channel; LR, the Foot Jueyin Liver Channel; LU, the Hand Taiyin Lung Channel; m30s STS, number of modified 30 sit-to-stand; MMSE, Mini Mental State Examination; MMT, Manual Muscle Test; NRS, numerical rating scale; NRS, numerical rating scale; PC, the Hand Jueyin pericardium Channel; PCA, patient-controlled analgesia; POCD, postoperative cognitive dysfunction; PPT, value of pressure pain threshold; QOL, quality of life; SDS, Self-Rating Depression Scale; SDS, self-rating depression scale; SF-MPQ, Short-Form McGill Pain Questionnaire; SJ, the Hand Shaoyang San jiao Channel; SP, the Foot Taiyin Spleen Channel; SRSS, Self-rating scale of sleep; ST, the Foot Yangming Stomach Channel; VAS, Visual Analogue Scale; WOMAC, Western Ontario and McMaster Universities Osteoarthritis Index.

In conclusion, 12 studies used electroacupuncture as an intervention in the acupuncture group^37,38,40,46–50,55,56,60,62^. In the remaining studies, five used conventional acupuncture^35,50,52,53,59^, four used auricular acupressure^36,38,42,58^, four applied moxibustion^39,41,54,61^, and one used warm acupuncture^45^. Laser moxibustion^43,44^ and dry needle^51^ were analyzed as special acupuncture together.

### Risk of bias

**Figure 2:**
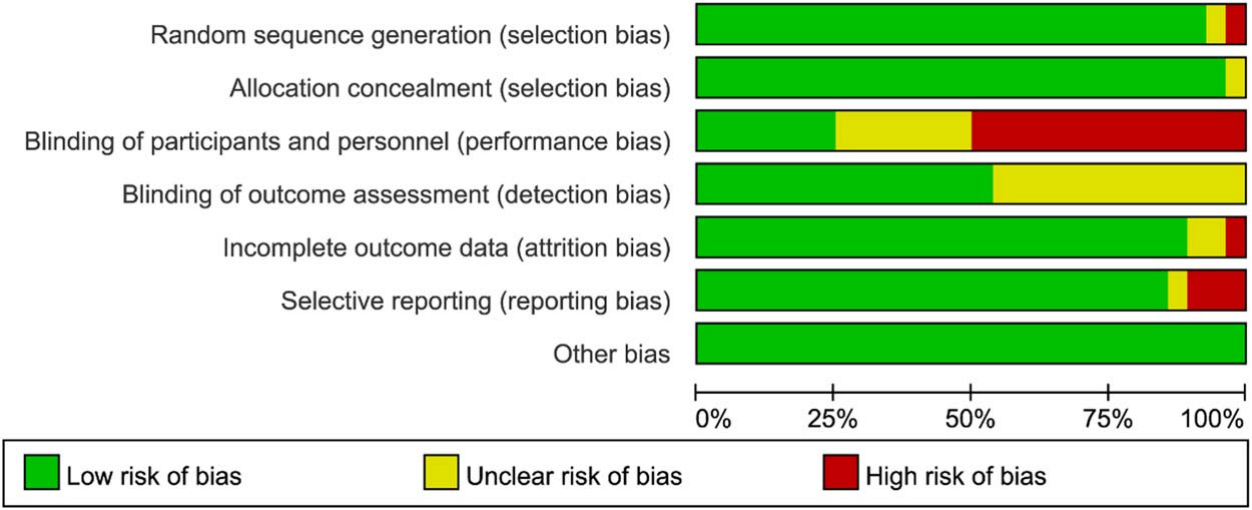
Risk of bias graph.

**Figure 3:**
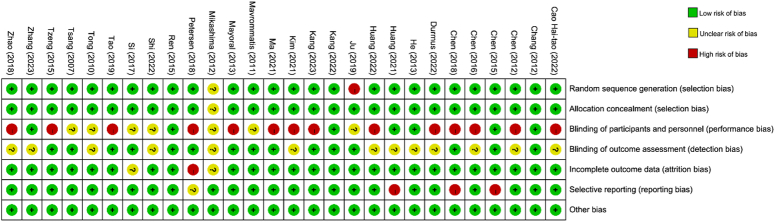
Risk of bias summary.

The summary of the risk of bias assessment across all included studies is presented in Figure 2. Blinding of participants and personnel (performance bias) (50%) was the most influential source of high risk of bias. Blinding of outcome assessment (50%), selective reporting (14%), and incomplete outcome data (11%) were also common sources of bias. Detailed information regarding the risk of bias for the included studies is shown in Figure 3.

### Network meta-analysis

Among the 28 studies included in the analysis, a total of 16 used the Visual Analogue Score (VAS) as an outcome indicator; HSS and ROM scores were used as outcome indicators in four studies, respectively. In addition, WOMAC stiffness scores were used in three studies. Therefore, the VAS score was used as the main outcome index for this NMA analysis, while HSS, ROM, and WOMAC stiffness were used as secondary outcome indexes. Among the literature included in the NMA, there were seven interventions: A represents conventional acupuncture, B represents electroacupuncture, C represents auricular acupressure, D represents warm acupuncture, E represents moxibustion, F represents special acupuncture (e.g. laser acupuncture, dry needling, etc.), G represents sham acupuncture (e.g. without needle penetrating skin), and H represents no acupuncture.

#### Primary outcomes

**Figure 4:**
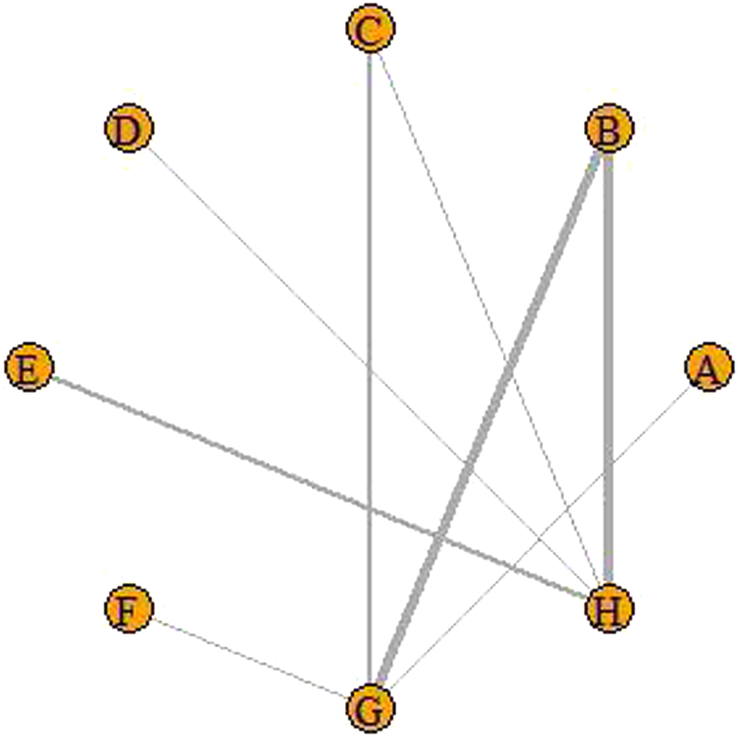
Network of the VAS scores of the selected studies. A, conventional acupuncture; B, electroacupuncture; C, auricular acupressure; D, warm acupuncture; E, moxibustion; F, special acupuncture; G, sham acupuncture; H, no acupuncture.

A total of 16 RCTs included VAS, forming an intervention node centered on electroacupuncture, involving all seven interventions. The network relationship of specific VAS is shown in Figure 4. The size of the dots in the graph represents the number of interventions, and the larger the dots, the greater the number of studies that have adopted the intervention. The thickness of the lines in the graph represents the number of studies involving the comparison of the corresponding two interventions, and the thicker the lines are, the stronger the association between the two interventions.

As the clinical efficacy index was the two-arm study, heterogeneity analysis was conducted. The heterogeneity test of NMA showed that the overall *I* ^2^>50%, indicating that there was significant statistical heterogeneity among the studies, so the random effects model was used for analysis.

**Figure 5:**
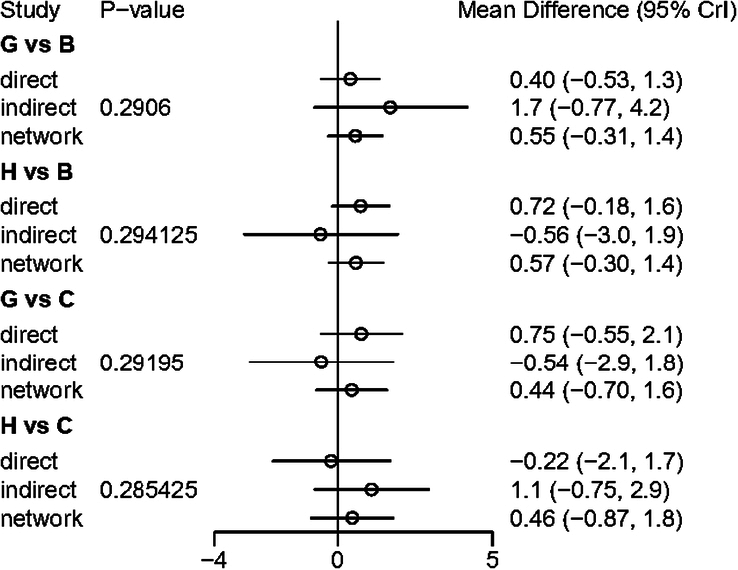
Inconsistency test of VAS scores. B, electroacupuncture; C, auricular acupressure; G, sham acupuncture; H, no acupuncture.

The results of the node separation method in VAS were all *P*>0.05, as shown in Figure 5. The data showed that there was no significant inconsistency between direct and indirect comparison of intervention and treatment measures.

**Figure 6:**
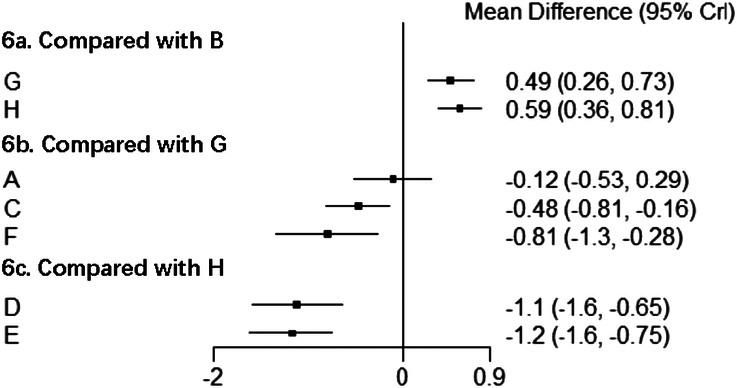
Forest plot for direct comparison of VAS scores. A, conventional acupuncture; B, electroacupuncture; C, auricular acupressure; D, warm acupuncture; E, moxibustion; F, special acupuncture; G, sham acupuncture; H, no acupuncture.

**Figure 7:**
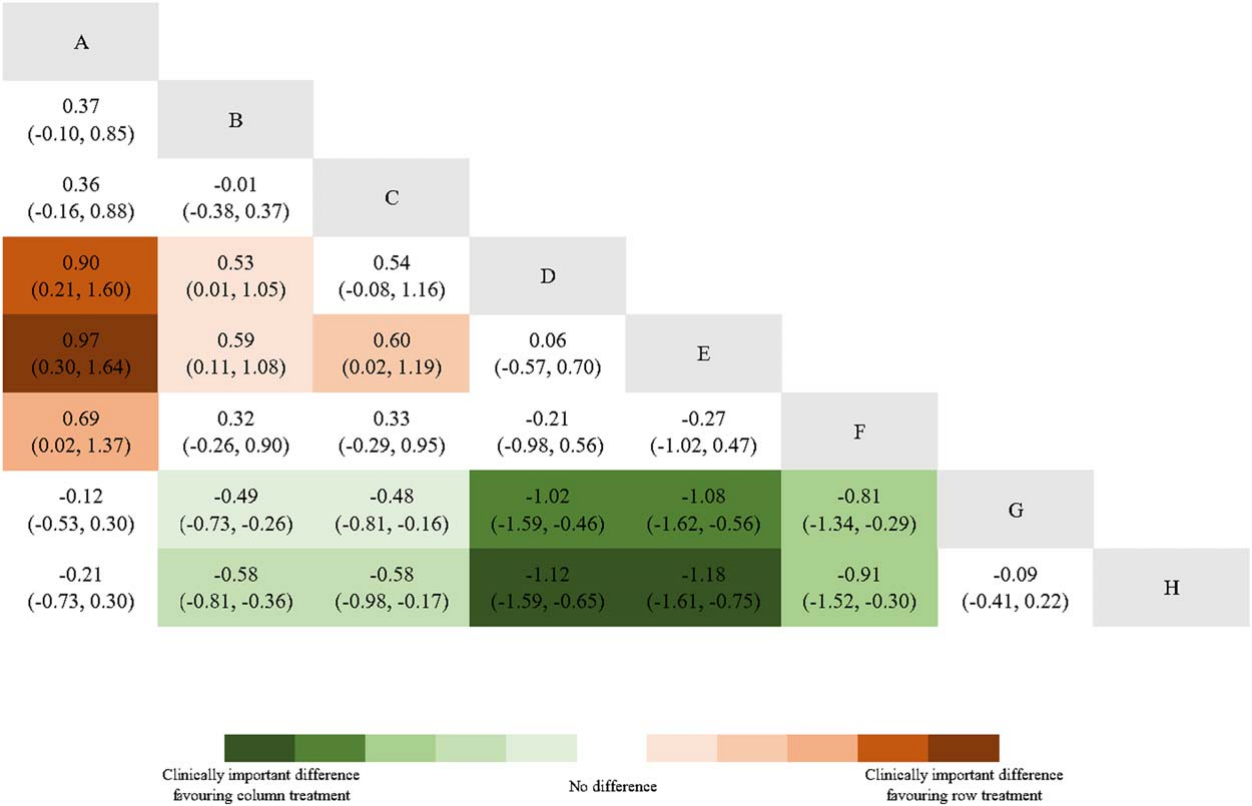
Relative effect estimates with 95% credible intervals of all interventions in VAS scores. A, conventional acupuncture; B, electroacupuncture; C, auricular acupressure; D, warm acupuncture; E, moxibustion; F, special acupuncture; G, sham acupuncture; H, no acupuncture. Green shading indicates that the intervention in the column is more effective than that in the row, while red shading shows that the intervention in the row is more effective. The intensity of the shading represents the magnitude of the treatment effect and its alignment with clinically significant outcomes.

**Figure 8:**
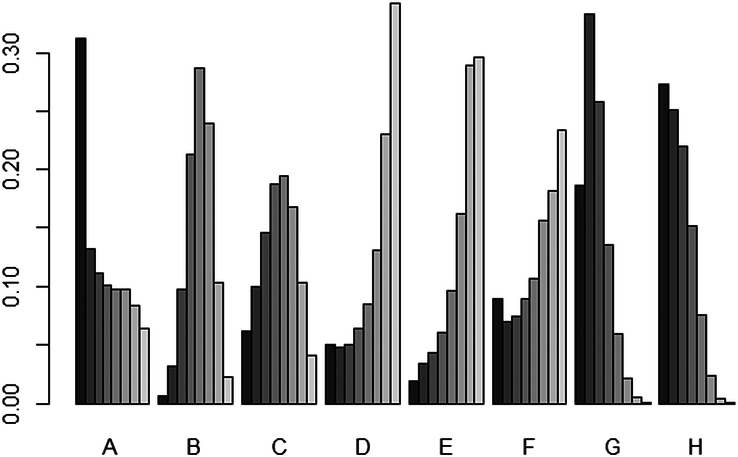
Ranking probability graph of all interventions in VAS scores. A, conventional acupuncture; B, electroacupuncture; C, auricular acupressure; D, warm acupuncture; E, moxibustion; F, special acupuncture; G, sham acupuncture; H, no acupuncture. The color gradient from light to dark represents the ranking from first to last, and taller bars indicate a higher probability of achieving a higher rank.

VAS score was evaluated using the consistency model for NMA (Fig. 6), and a relative effect matrix was additionally created for all comparisons between the interventions (Fig. 7). Figure 8 represents the ranking probability of each intervention in the form of a bar chart. The results indicated that five acupuncture-related therapies other than conventional acupuncture can effectively relieve the pain after TKA. The results suggest that the VAS scores of electroacupuncture, auricular acupressure, warm acupuncture, moxibustion, and special acupuncture were obviously lower than those of sham acupuncture and conventional rehabilitation therapy without acupuncture. Besides, among the acupuncture-related interventions, warm acupuncture and moxibustion performed better than conventional acupuncture and electroacupuncture. There was no significant difference in other comparisons.

#### Secondary outcomes

##### Results of NMA of HSS scores

Among all the 28 studies included, four studies reported HSS scores as outcome indicators. After examining the network plot of HSS scores, it was found that there were unconnected nodes in the network or there was not enough comparative data to connect all nodes, so the “Gemtc” package was not able to create a model. Therefore, the data and conclusions of these four studies were analyzed separately by manual review.

The four studies respectively applied moxibustion^39^, auricular acupressure^42^, and electroacupuncture^37,49^ as the treatment of the intervention group. Three out of these four studies employed no acupuncture in the control group, while one employed sham acupuncture. All of these studies concluded that acupuncture-related therapies were efficient for the rehabilitation of TKA patients.

##### Results of NMA of ROM scores

**Figure 9:**
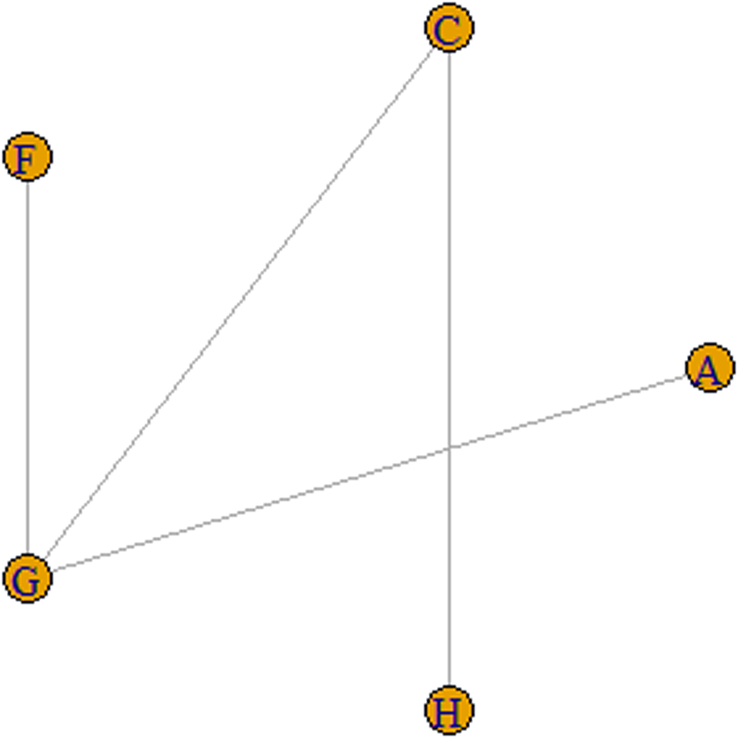
Network of the ROM scores of the selected studies. A, conventional acupuncture; C, auricular acupressure; F, special acupuncture; G, sham acupuncture; H, no acupuncture.

Four studies included ROM score in outcome indicators involving auricular acupressure^42,58^, conventional acupuncture^35^, and special acupuncture (i.e. dry needling)^51^. The network relationship of ROM scores after the use of different interventions is shown in Figure 9.

**Figure 10:**
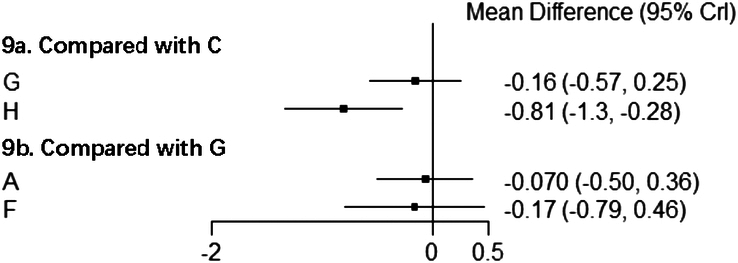
Forest plot for direct comparison of ROM scores. A, conventional acupuncture; C, auricular acupressure; F, special acupuncture; G, sham acupuncture; H, no acupuncture.

According to the relative effect of different interventions, auricular acupressure can significantly decrease the ROM score compared to no acupuncture, while the ROM score differences between the other interventions showed no significant meaning (Fig. 10).

##### Results of NMA of WOMAC stiffness scores

**Figure 11:**
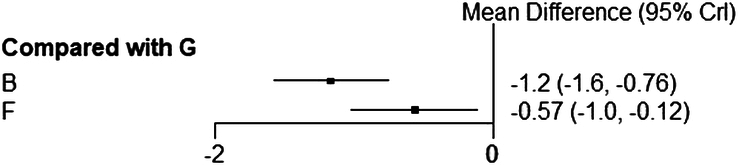
Forest plot for direct comparison of WOMAC stiffness scores. B, electroacupuncture; F, special acupuncture; G, sham acupuncture.

Three studies reported WOMAC scores, involving electroacupuncture^48,50^ and special acupuncture (i.e. laser acupuncture)^43^. However, based on the reporting bias, only WOMAC stiffness scores were extracted and included in the analysis. Compared with sham acupuncture, both electroacupuncture and special acupuncture had a more significant effect on lowering the WOMAC stiffness score, and electroacupuncture performed better than special acupuncture (Fig. 11).

### Adverse events

Of the 28 RCTs included in the analysis, a total of 18 articles reported the condition of adverse events in patients after acupuncture intervention. Among them, nine articles indicated no adverse reaction, and those reported to have occurred in the remaining nine articles included minor pain and bleeding, vomiting, nausea, dizziness, etc. The specific number of cases of each reaction is shown in Table 2. The overall adverse reactions were mild, and all of them were common symptoms after TKA^65^. Therefore, it can be generally concluded that acupuncture-related therapies are safe and reliable for postoperative rehabilitation of TKA, but it also suggests that clinical practitioners should pay full attention to the patient’s reactions and healing during the implementation.

### Publish bias

**Figure 12:**
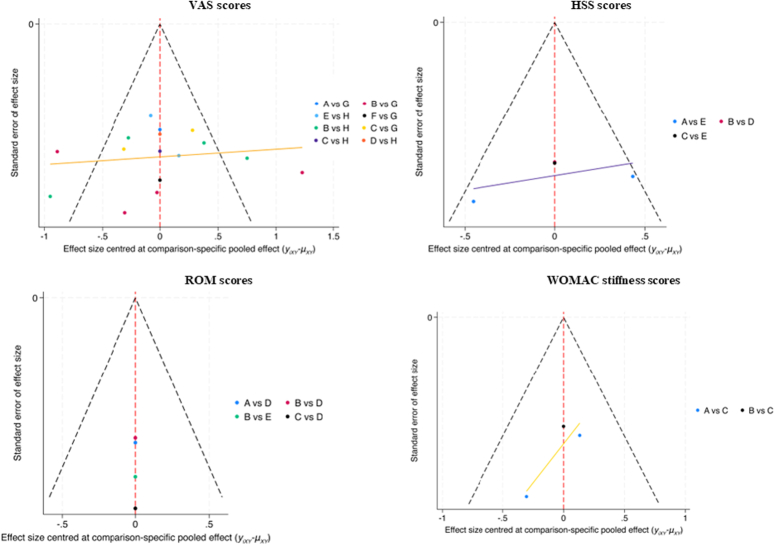
Funnel plot of the included papers. A, conventional acupuncture; B, electroacupuncture; C, auricular acupressure; D, warm acupuncture; E, moxibustion; F, special acupuncture; G, sham acupuncture; H, no acupuncture.

A publication bias test on the included RCTs was conducted and illustrated with a corrected comparison funnel plot (Fig. 12). The symmetry of the four funnel plots was poor. Most scatter points were located in the lower-middle region, and four studies in the VAS score funnel plot fell outside the 95% CI, indicating potential publication bias or small sample effects. After careful data analysis, it was determined that this bias was not due to clinical methods or statistical issues. Since these studies met the inclusion criteria, they were retained in the statistical analysis.

## Discussion

This study presented the inaugural network meta-analysis (NMA) aimed at identifying the most effective form of acupuncture intervention for postoperative rehabilitation in patients who have undergone TKA. The analysis included data from 28 randomized controlled trials involving a total of 2128 participants. Various acupuncture-related therapies commonly employed in TKA patients were compared, with a specific focus on pain relief, range of motion, inflammatory status, and joint stiffness. Among the seven distinct acupuncture-related interventions examined, the study found that electroacupuncture yielded the most pronounced improvements in pain relief, followed by special acupuncture and auricular acupressure. Auricular acupressure demonstrated a significant positive effect on motor function, while electroacupuncture showed superior efficacy in reducing the inflammation index compared to special acupuncture.

This study highlighted the analgesic effects of acupuncture methods such as auricular acupressure, needle acupuncture, and electroacupuncture. Auricular acupressure, based on traditional Chinese medicine principles, is widely used as a complementary healthcare strategy. The external ear, which resembles an inverted fetus, exhibits rich innervation and complex neural networks^66,67^. Stimulating specific areas of the ear can regulate corresponding body parts and alleviate pain by increasing the secretion of endorphins and serotonin, inhibiting pain signal transmission, and reducing pain perception^68^.

In this study, four main acupoints for ear acupressure were identified: Shenmen, Lung, Subcortex, and Knee Joint. Preoperative auricular acupressure has been shown to reduce the occurrence of pain-related adverse reactions in TKA patients^69^. Acupuncture provides short-term pain relief for patients undergoing total knee replacement, consistent with findings from Kelly and Willis^70^.

Acupuncture’s mechanisms of action involve various biological responses^71^, including the stimulation of nerve impulses and the release of neurotransmitters, which modulate the function of the brain and peripheral system^72,73^. One of the most well-known mechanisms is through endogenous opioids and their receptors. Early research^74,75^ indicated the involvement of endogenous central nervous system opioids in acupuncture analgesia. Different types of endogenous opioids, such as beta-endorphin, have been reported as frequency-dependent factors in acupuncture. Studies^76,77^ on the mechanisms of action have shown that endogenous opioid peptides in the central nervous system play a significant role in mediating the analgesic effects of electroacupuncture. The frequency of electroacupuncture ranges from 2 to 100 Hz. Similarly, Lin and Chen^78^ reported the pain-relieving effects of triggering enkephalin (Enk) and releasing Enk and endorphins (EM) using 2 Hz electroacupuncture, as well as triggering dynorphin (Dyn) using 100 Hz electroacupuncture. Electroacupuncture with an intensity of 2 mA and a frequency of 2 Hz has also been used for post-TKA pain relief^79^. The NMA of the study results demonstrates the analgesic effects of acupuncture. It is hoped that acupuncture intervention can become a choice for postoperative pain management in patients.

### Clinical implications

The findings of this study have important clinical implications, suggesting that acupuncture intervention can effectively alleviate postoperative pain and contribute to reduced analgesic drug dosages. Preoperative ear acupuncture conducted once prior to surgery has demonstrated the potential to minimize adverse reactions associated with analgesia administration^42^. Key acupoints for both acupuncture and electroacupuncture include Xuehai (SP10), Liangqiu (ST34), Dubi (ST35), Neixiyan (EX-LE4), Yanglingquan (GB34), and Zusanli (ST36). Electroacupuncture is typically administered under general anesthesia, utilizing a frequency range of 2–100 Hz. It is recommended to consider incorporating acupuncture treatment alongside routine analgesic drug usage for postoperative patients. While acupuncture is traditionally performed by medical professionals, nurses can also provide acupressure on designated points after acquiring knowledge of pain relief locations or perform ear acupressure under the supervision of a doctor. This approach can enhance patient comfort and allow nurses to deliver specialized care while fulfilling their professional responsibilities.

### Research implications

The present literature review findings indicate that the majority of studies demonstrate significant analgesic effects of acupuncture, with most studies utilizing randomized controlled trial designs. However, the measurement tools used primarily consisted of various pain assessment scales, while physiological indicators such as hemodynamics and autonomic nervous system responses were not included. This omission may have influenced the observed analgesic effects in some studies. Furthermore, the analysis suggests that the inclusion of sham acupuncture groups in the experimental design did not significantly impact the reliability of the empirical results, thus clarifying the specific analgesic effects of acupuncture.

### Limitations

This study encompassed 28 RCTs primarily conducted in Eastern regions, with the Chinese mainland and Taiwan region serving as the main regions of origin. The studies included in this analysis were predominantly published in Chinese and English. Further trials with various cultural backgrounds are needed to construct a comprehensive literature base, which would facilitate a more thorough acupuncture rehabilitation after TKA.

Given the unique nature of acupuncture intervention, achieving fully double-blind designs in the majority of the included RCTs proved challenging. Although considerable efforts were made by many studies to ensure participant blinding, the inherent variability in sensations experienced by patients receiving different acupuncture techniques, as well as the necessary knowledge possessed by practitioners to perform the acupuncture procedures, unavoidably introduce a certain degree of bias. However, it is crucial to recognize that such limitations are inherent to acupuncture interventions themselves and are, therefore, genuinely unavoidable. Additionally, it should be noted that acupuncture can have added psychological effects on patients undergoing TKA, which may positively influence treatment outcomes. Consequently, this inherent bias is unlikely to fundamentally impact the overall conclusions drawn from the study.

## Conclusion

Acupuncture intervention, especially electroacupuncture, can effectively alleviate pain, improve motion, reduce stiffness, and decrease inflammation in post-TKA patients with no serious adverse events.

## Ethical approval

Not applicable.

## Consent

Not applicable.

## Source of funding

Not applicable.

## Author contribution

W.-X.L.: investigation, data curation, formal analysis, and writing of the original draft; C.-Q.W. and W.F.: investigation, data curation, and formal analysis; Y.-J.Z.: writing – review and editing; L.Y. and H.-J.J.: formal analysis and data curation; J.P.: conceptualization, methodology, data curation, and writing – review and editing; K.-P.L.: conceptualization, methodology, data curation, writing – review and editing, and supervision. All authors agree to be accountable for all aspects of this work.

## Conflicts of interest disclosure

The authors declare no conflicts of interest.

## Research registration unique identifying number (UIN)

The study protocol was pre-registered in the PROSPERO database (CRD42023435482) and implemented in strict adherence to the guidelines outlined in the PRISMA extension statement for network meta-analysis.

## Guarantor

Kun-Peng Li, School of Exercise and Health, Shanghai University of Sport, Shanghai 200438, People’s Republic of China; e-mail: xyfyli@163.com.

## Data availability statement

The datasets used and/or analyzed during the current study are available from the corresponding author on reasonable request.

## Provenance and peer review

Not commissioned, externally peer-reviewed.

## Supplementary Material
