# Supplementary material for: Acupuncture for rehabilitation after total knee arthroplasty: a systematic review and network meta-analysis
Source: Int J Surg. 2024 Aug 5;111(1):1373–85. doi: 10.1097/JS9.0000000000002006 (PMC11745769; doi:10.1097/JS9.0000000000002006)

Pubmed 69

<https://pubmed.ncbi.nlm.nih.gov/?term=longquerya7bececa3eee30ac745a&filter=pubt.clinicaltrial&filter=pubt.randomizedcontrolledtrial&ac=no&sort=relevance>

(("arthroplasty, replacement, knee"[MeSH Terms] OR ("arthroplasty knee replacement"[Title/Abstract] OR "knee replacement arthroplast*"[Title/Abstract] OR (("replace"[All Fields] OR "replaceable"[All Fields] OR "replaced"[All Fields] OR "replaces"[All Fields] OR "replacing"[All Fields] OR "replacment"[All Fields] OR "replantation"[MeSH Terms] OR "replantation"[All Fields] OR "Replacement"[All Fields] OR "replacements"[All Fields]) AND "arthroplasties knee"[Title/Abstract]) OR "knee arthroplasty total"[Title/Abstract] OR "arthroplasty total knee"[Title/Abstract] OR "total knee arthroplasty"[Title/Abstract] OR "replacement total knee"[Title/Abstract] OR "total knee replacement"[Title/Abstract] OR "knee replacement total"[Title/Abstract] OR "knee arthroplasty"[Title/Abstract] OR "arthroplasty knee"[Title/Abstract] OR (("Arthroplasty"[MeSH Terms] OR "Arthroplasty"[All Fields] OR "Arthroplasties"[All Fields]) AND "knee replacement"[Title/Abstract]) OR (("replace"[All Fields] OR "replaceable"[All Fields] OR "replaced"[All Fields] OR "replaces"[All Fields] OR "replacing"[All Fields] OR "replacment"[All Fields] OR "replantation"[MeSH Terms] OR "replantation"[All Fields] OR "Replacement"[All Fields] OR "replacements"[All Fields]) AND "arthroplasty knee"[Title/Abstract]) OR (("arthroplasty, replacement"[MeSH Terms] OR ("Arthroplasty"[All Fields] AND "Replacement"[All Fields]) OR "replacement arthroplasty"[All Fields] OR ("Arthroplasty"[All Fields] AND "Replacement"[All Fields]) OR "arthroplasty replacement"[All Fields]) AND "partial knee"[Title/Abstract]) OR "unicompartmental knee arthroplasty"[Title/Abstract] OR "arthroplasty unicompartmental knee"[Title/Abstract] OR "knee arthroplasty unicompartmental"[Title/Abstract] OR "unicondylar knee arthroplasty"[Title/Abstract] OR (("Arthroplasty"[MeSH Terms] OR "Arthroplasty"[All Fields] OR "Arthroplasties"[All Fields]) AND "unicondylar knee"[Title/Abstract]) OR "knee arthroplasty unicondylar"[Title/Abstract] OR "partial knee arthroplasty"[Title/Abstract] OR "arthroplasty partial knee"[Title/Abstract] OR "knee arthroplasty partial"[Title/Abstract] OR "unicondylar knee replacement"[Title/Abstract] OR "knee replacement unicondylar"[Title/Abstract] OR "partial knee replacement"[Title/Abstract] OR "knee replacement partial"[Title/Abstract] OR "unicompartmental knee replacement"[Title/Abstract] OR ("arthroplasty, replacement, knee"[MeSH Terms] OR ("Arthroplasty"[All Fields] AND "Replacement"[All Fields] AND "Knee"[All Fields]) OR "knee replacement arthroplasty"[All Fields] OR ("Knee"[All Fields] AND "Replacement"[All Fields] AND "Unicompartmental"[All Fields]) OR "knee replacement unicompartmental"[All Fields]))) AND ("acupunctural"[All Fields] OR "acupuncture"[MeSH Terms] OR "acupuncture"[All Fields] OR "acupuncture therapy"[MeSH Terms] OR ("acupuncture"[All Fields] AND "therapy"[All Fields]) OR "acupuncture therapy"[All Fields] OR "acupuncture s"[All Fields] OR "acupunctured"[All Fields] OR "acupunctures"[All Fields] OR "acupuncturing"[All Fields] OR ("acupuncture"[MeSH Terms] OR "acupuncture"[All Fields] OR "pharmacopuncture"[All Fields] OR "pharmacopunctures"[All Fields]) OR ("electroacupuncture"[MeSH Terms] OR "electroacupuncture"[All Fields] OR "electroacupuncturing"[All Fields]) OR "acup*"[All Fields] OR ("transcutanclus"[All Fields] AND ("electricity"[MeSH Terms] OR "electricity"[All Fields] OR "electric"[All Fields] OR "electrical"[All Fields] OR "electrically"[All Fields] OR "electrics"[All Fields]) AND ("acupoint s"[All Fields] OR "acupuncture points"[MeSH Terms] OR ("acupuncture"[All Fields] AND "points"[All Fields]) OR "acupuncture points"[All Fields] OR "acupoint"[All Fields] OR "acupoints"[All Fields]) AND ("stimulate"[All Fields] OR "stimulated"[All Fields] OR "stimulates"[All Fields] OR "stimulating"[All Fields] OR "stimulation"[All Fields] OR "stimulations"[All Fields] OR "stimulative"[All Fields] OR "stimulator"[All Fields] OR "stimulator s"[All Fields] OR "stimulators"[All Fields])) OR ("needle s"[All Fields] OR "needled"[All Fields] OR "needles"[MeSH Terms] OR "needles"[All Fields] OR "needle"[All Fields] OR "needling"[All Fields] OR "needlings"[All Fields]) OR ("moxibustion"[MeSH Terms] OR "moxibustion"[All Fields]))) AND (clinicaltrial[Filter] OR randomizedcontrolledtrial[Filter])


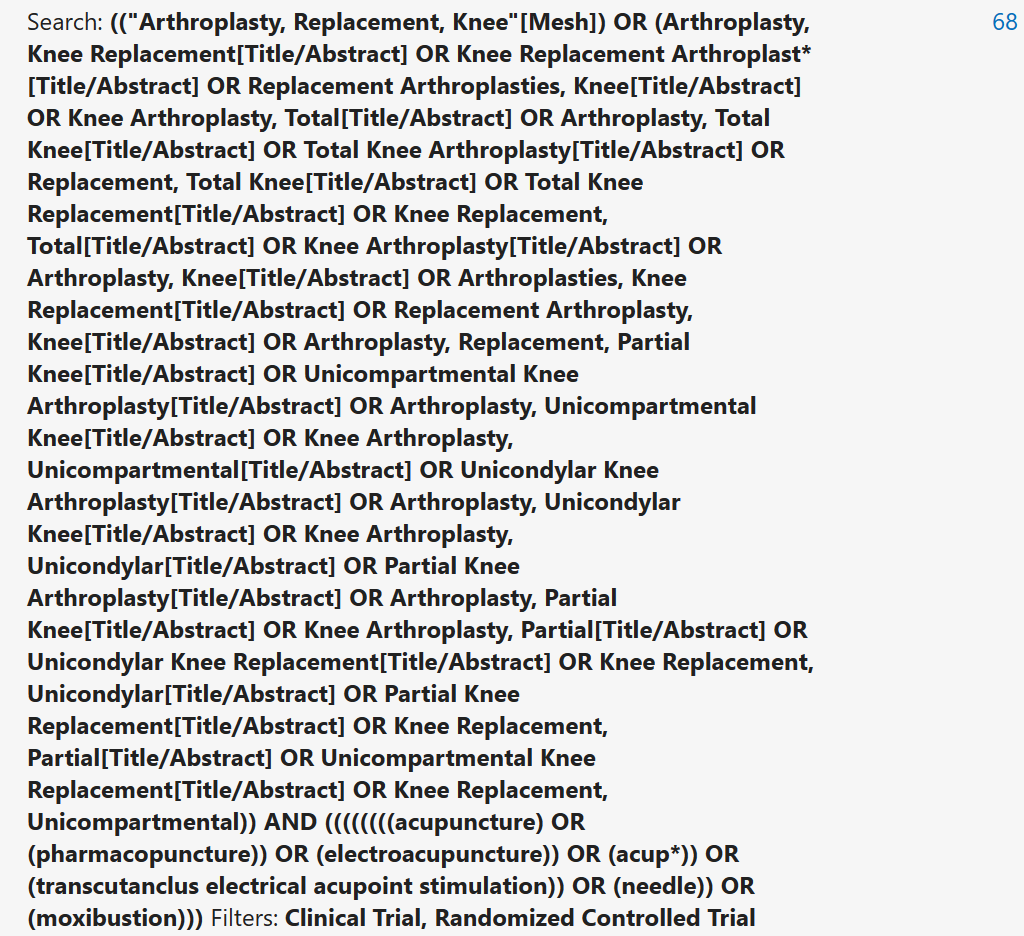


WOS 93

<https://www.webofscience.com/wos/alldb/summary/b3bc7292-588d-44b4-82bb-d3cc3acd66e8-7b08bf9a/relevance/1>

Scopus 132

TITLE-ABS-KEY("acupuncture" OR "pharmacopuncture" OR "electroacupuncture" OR "acup*" OR "transcutanclus electrical acupoint stimulation" OR "needle" OR "moxibustion") AND TITLE-ABS-KEY("arthroplasty, replacement, knee" OR "knee replacement arthroplast*" OR "replacement arthroplasties, knee" OR "knee arthroplasty, total" OR "Arthroplasty, Total Knee" OR "Total Knee Arthroplasty" OR "Replacement, Total Knee" OR "Total Knee Replacement" OR "Knee Replacement, Total" OR "Knee Arthroplasty" OR "Arthroplasty, Knee" OR "Arthroplasties, Knee Replacement" OR "Replacement Arthroplasty, Knee" OR "Arthroplasty, Replacement, Partial Knee" OR "Unicompartmental Knee Arthroplasty" OR "Arthroplasty, Unicompartmental Knee" OR "Knee Arthroplasty, Unicompartmental" OR "Unicondylar Knee Arthroplasty" OR "Arthroplasty, Unicondylar Knee" OR "Knee Arthroplasty, Unicondylar" OR "Partial Knee Arthroplasty" OR "Arthroplasty, Partial Knee" OR "Knee Arthroplasty, Partial" OR "Unicondylar Knee Replacement" OR "Knee Replacement, Unicondylar" OR "Partial Knee Replacement" OR "Knee Replacement, Partial" OR "Unicompartmental Knee Replacement" OR "Knee Replacement, Unicompartmental”) AND TITLE-ABS-KEY("randomized controlled trial")

Embase 108

('acupuncture':ab,ti OR 'pharmacopuncture':ab,ti OR 'electroacupuncture':ab,ti OR 'acup*':ab,ti OR 'teas':ab,ti OR 'needle':ab,ti OR 'moxibustion':ab,ti) AND ('arthroplasties, replacement, knee':ab,ti OR 'arthroplasty, knee replacement':ab,ti OR 'knee replacement arthroplasties':ab,ti OR 'knee replacement arthroplasty':ab,ti OR 'replacement arthroplasties, knee':ab,ti OR 'knee arthroplasty, total':ab,ti OR 'arthroplasty, total knee':ab,ti OR 'total knee arthroplasty':ab,ti OR 'replacement, total knee':ab,ti OR 'total knee replacement':ab,ti OR 'knee replacement, total':ab,ti OR 'knee arthroplasty':ab,ti OR 'arthroplasty, knee':ab,ti OR 'arthroplasties, knee replacement':ab,ti OR 'replacement arthroplasty, knee':ab,ti OR 'arthroplasty, replacement, partial knee':ab,ti OR 'unicompartmental knee arthroplasty':ab,ti OR 'arthroplasty, unicompartmental knee':ab,ti OR 'knee arthroplasty, unicompartmental':ab,ti OR 'unicondylar knee arthroplasty':ab,ti OR 'arthroplasty, unicondylar knee':ab,ti OR 'knee arthroplasty, unicondylar':ab,ti OR 'partial knee arthroplasty':ab,ti OR 'arthroplasty, partial knee':ab,ti OR 'knee arthroplasty, partial':ab,ti OR 'unicondylar knee replacement':ab,ti OR 'knee replacement, unicondylar':ab,ti OR 'partial knee replacement':ab,ti OR 'knee replacement, partial':ab,ti OR 'unicompartmental knee replacement':ab,ti OR 'knee replacement, unicompartmental':ab,ti)


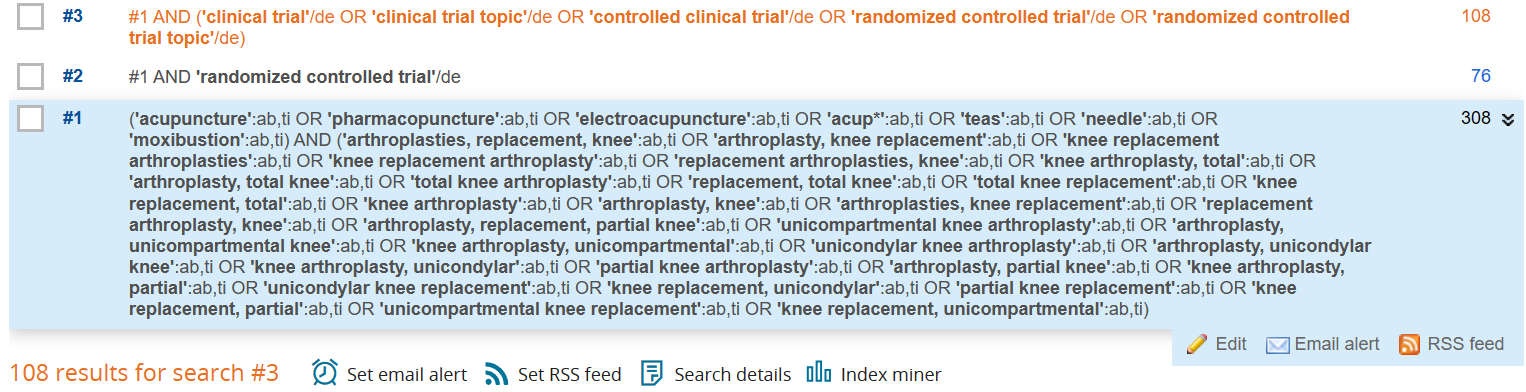


Cochrane 134

(Arthroplasties, Replacement, Knee):ti,ab,kw OR (Arthroplasty, Knee Replacement):ti,ab,kw OR (Knee Replacement Arthroplasties):ti,ab,kw OR (Knee Replacement Arthroplasty):ti,ab,kw OR (Replacement Arthroplasties, Knee):ti,ab,kw OR (Knee Arthroplasty, Total):ti,ab,kw OR (Arthroplasty, Total Knee):ti,ab,kw OR (Total Knee Arthroplasty):ti,ab,kw OR (Replacement, Total Knee):ti,ab,kw OR (Total Knee Replacement):ti,ab,kw OR (Knee Replacement, Total):ti,ab,kw OR (Knee Arthroplasty Arthroplasty, Knee):ti,ab,kw OR (Arthroplasties, Knee Replacement):ti,ab,kw OR (Replacement Arthroplasty, Knee):ti,ab,kw OR (Arthroplasty, Replacement, Partial Knee):ti,ab,kw OR (Unicompartmental Knee Arthroplasty):ti,ab,kw OR (Arthroplasty, Unicompartmental Knee):ti,ab,kw OR (Knee Arthroplasty, Unicompartmental):ti,ab,kw OR (Unicondylar Knee Arthroplasty):ti,ab,kw OR (Arthroplasty, Unicondylar Knee):ti,ab,kw OR (Knee Arthroplasty, Unicondylar):ti,ab,kw OR (Partial Knee Arthroplasty):ti,ab,kw OR (Arthroplasty, Partial Knee):ti,ab,kw OR (Knee Arthroplasty, Partial):ti,ab,kw OR (Unicondylar Knee Replacement):ti,ab,kw OR (Knee Replacement, Unicondylar):ti,ab,kw OR (Partial Knee Replacement):ti,ab,kw OR (Knee Replacement, Partial):ti,ab,kw OR (Unicompartmental Knee Replacement):ti,ab,kw OR (Knee Replacement, Unicompartmental):ti,ab,kw

(acupuncture):ti,ab,kw OR (pharmacopuncture):ti,ab,kw OR (electroacupuncture):ti,ab,kw OR (acup*):ti,ab,kw OR (transcutanclus electrical acupoint stimulation):ti,ab,kw(needle):ti,ab,kw OR (moxibustion):ti,ab,kw

(randomised controlled trial):ti,ab,kw OR (RCT):ti,ab,kw


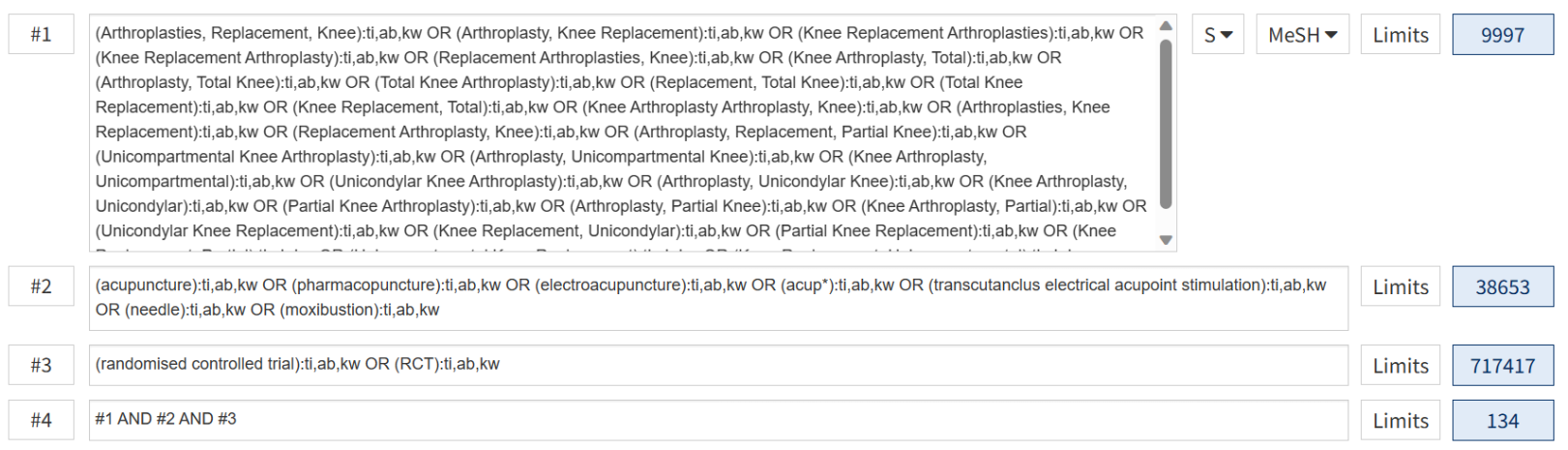


Clinicaltrials.gov 12

<https://clinicaltrials.gov/ct2/results?cond=Arthroplasty%2C+Knee%2C+Replacement&term=&type=Intr&rslt=With&recrs=e&recrs=c&age_v=&age=0&age=1&age=2&gndr=&hlth=Y&intr=&titles=&outc=&spons=&lead=&id=&cntry=&state=&city=&dist=&locn=&rsub=&strd_s=&strd_e=&prcd_s=&prcd_e=&sfpd_s=&sfpd_e=&rfpd_s=&rfpd_e=&lupd_s=&lupd_e=&sort=>


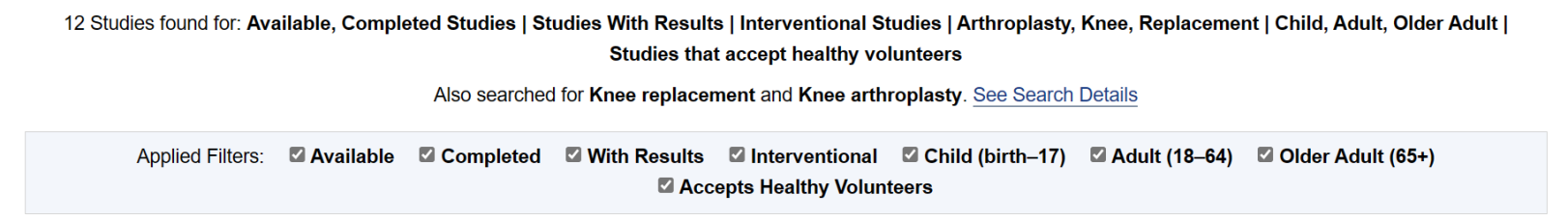

Supplement: Supplementary file 3 [file js9-111-1373-s003.docx]
