# Supplementary material for: Acupuncture for rehabilitation after total knee arthroplasty: a systematic review and network meta-analysis
Source: Int J Surg. 2024 Aug 5;111(1):1373–85. doi: 10.1097/JS9.0000000000002006 (PMC11745769; doi:10.1097/JS9.0000000000002006)
Supplement: Supplementary file 4 [file js9-111-1373-s004.pdf]

```
library("gemtc")
library("rjags")
library("coda")
library("brms")
library("readxl")

data <- read.csv("WOMAC stiffness_SMD_R.csv", sep=",", header=T)
network <- mtc.network(data.re = data)
summary(network)
plot(network)

model<-mtc.model(network,type = "consistency",n.chain =
3,likelihood="normal",link="identity",linearModel="random")
results <-mtc.run(model,n.adapt = 5000, n.iter = 20000, thin = 1)
summary(results)
plot(results)
gemtc::forest(results)

styles <- data.frame(
  style=c('normal', 'pooled', 'group'),
  font.weight=c('plain', 'plain', 'bold'),
  row.height=c(1, 1, 1.5),
  pe.style=c('square', 'square', NA),
  pe.scale=c(FALSE, FALSE, NA),
  lty=c(1,1,NA))
rownames(styles) <- styles[['style']]
gemtc::forest(results, styles=styles)
```
